# Multiple rectal carcinoids with diffuse ganglioneuromatosis

**DOI:** 10.1186/1477-7819-5-19

**Published:** 2007-02-16

**Authors:** Masashi Haraguchi, Hideki Kinoshita, Miho Koori, Noritsugu Tsuneoka, Taiichiro Kosaka, Yuji Ito, Junichiro Furui, Takashi Kanematsu

**Affiliations:** 1 Department of Surgery, Goto Central Hospital, Nagasaki, Japan; 2 Department of Internal Medicine, Goto Central Hospital, Nagasaki, Japan; 3 Department of Pathology, SRL Nishi-nihon, Japan; 4 Department of Surgery, Graduate School of Biomedical Sciences, Nagasaki University, Japan

## Abstract

**Background:**

Rectal carcinoids comprise only about 1% of all anorectal neoplasms. In addition, ganglioneuroma of the gastrointestinal tract is a rare tumor composed ganglion cells, nerve fibers, and supporting cells. Multiple carcinoid tumors with diffuse ganglioneuromatosis limited to the rectum are quite unusual.

**Case presentation:**

A 69-year-old man was referred to us because of about 100 small submucosal rectal tumors. He underwent abdominoperineal resection. Pathology revealed carcinoid tumors for about 30 submucosal nodules and diffuse ganglioneuromotosis. To date (6 months later) he remains well with no recurrence.

**Conclusion:**

Although the optimal treatment for the multiple rectal carcinoids remains to be clearly established, it is believed that not all patients with multiple rectal carcinoids (measuring less than 1 cm in diameter) need to have a radical operation. However, the treatment plan for each case should be individualized and a careful follow-up is mandatory.

## Background

Carcinoid tumors were initially described as a morphologically distinct subset of small intestinal neoplasms with a less aggressive behavior than that of the intestinal adenocarcinoma. Carcinoid tumors of the rectum comprise only about 1% of all anorectal neoplasms [1]. Typically, rectal carcinoids present as small, solitary submucosal nodules. Multicentricity is an even more rare occurrence. Only 33 patients with multiple rectal carcinoids, including our patient, have so far been reported in Japan [2].

Gastrointestinal ganglioneuromas are also rare tumors that are generally well differentiated and benign tumors. They commonly occur in the retroperitoneum and posterior mediastinum. Though they may be found anywhere in the body, particularly in the distribution of the major sympathetic ganglia, their involvement in the gastrointestinal tract is a rare occurrence. Some reports have indicated that ganglioneuromas of the gastrointestinal tract have been found in patients with several systemic disorders including multiple endocrine neoplasia IIB (MEN IIB), von Recklinghausen's disease, tuberous sclerosis, Cowden's disease, juvenile polyposis, filiform polyposis, and colonic adenocarcinoma [3,4].

Multiple carcinoid tumors with diffuse ganglioneuromatosis limited to the rectum are quite unusual. The relationship between multiple carcinoid tumors and gastrointestinal ganglioneuromatosis of the rectum is herein discussed.

## Case presentation

**Figure 1:**
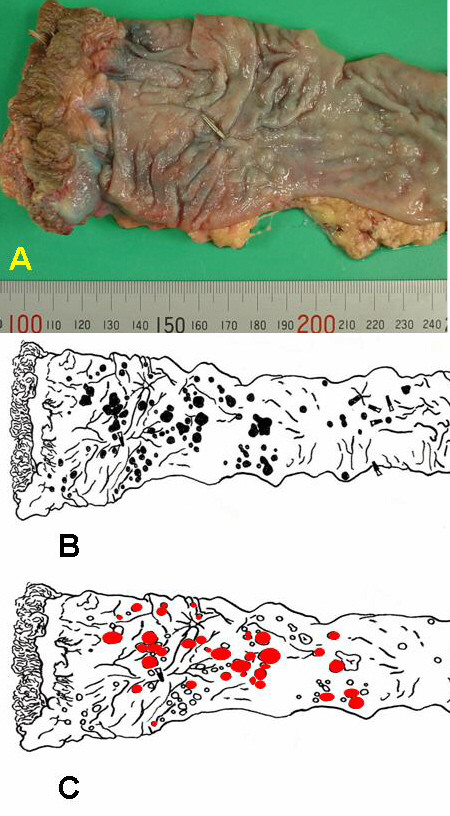
**a) **Macroscopic findings of the resected rectum demonstrating multiple submucosal tumors. **1.b) **A schematic drawing of the resected rectum showing the location of submucasal tumors (●).**1.c) **A schematic drawing of the resected rectum showing the location of carcinoid tumors ().

**Figure 2:**
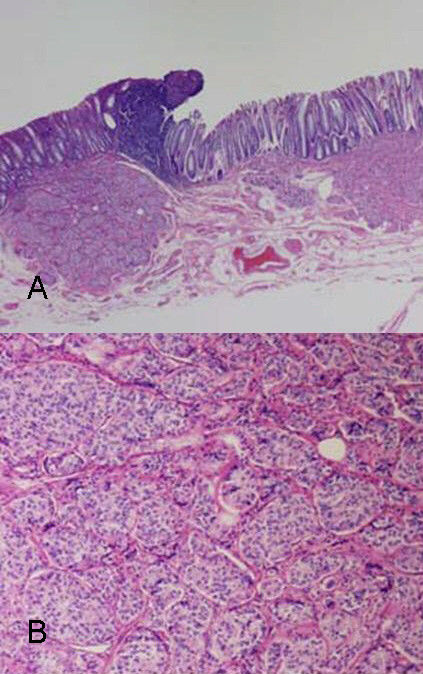
**a) **A carcinoid tumor proliferating submucosa **b) **A carcinoid tumor: uniform small round, polygonal prominent round nuclei.

**Figure 3:**
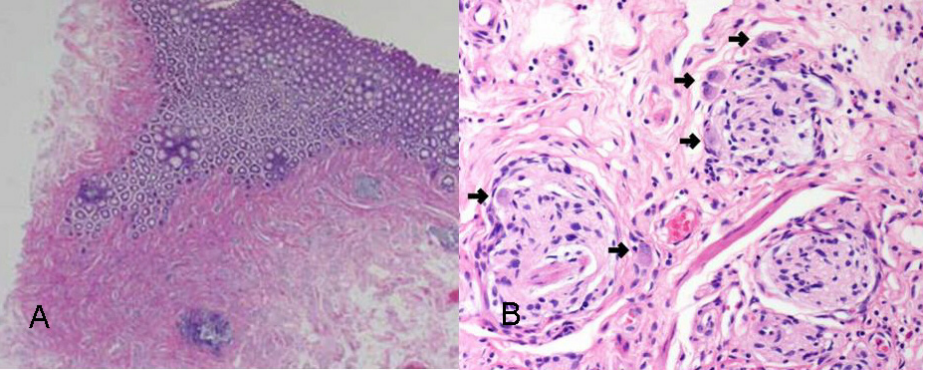
**a) **A section of the mucosa and submucosa showing extensive ganglioneuromatosis **b) **A ganglion cell (*arrow*) is surrounded by spindle cells.

A 69-year-old man was referred to us because of about 100 small submucosal rectal tumors detected at examination by his private physician. Multiple biopsies reported to be a tentative diagnosis of multiple carcinoid tumors. He had never been diagnosed as having multiple endocrine neoplasia (MEN) or other multiple tumor syndromes. His family history was not contributory. Physical examination revealed no abnormalities. Serum serotonin level was within normal range, 221 ng/ml. Tumor markers were within normal limits, CEA (carcinoembryonic antigen) 2.3 ng/ml, CA (carbohydrate antigen) 19-9 <2.0 U/ml. Computed tomographs of the brain, chest, abdomen and pelvis did not show any abnormality. He underwent abdominoperineal resection. Pathology revealed carcinoid tumors for about 30 submucosal nodules, which especially concentrated in the lower rectum (Figure 1a–c, Figure 2a,b) and diffuse ganglioneuromotosis (Figure 3a,b). Both carcinoid tumors and ganglioneuroma located within the mucosal and submucosal layer. There was neither metastasis to the liver nor the lymph node. The patient had an uneventful recovery and is maintaining good health at 6 months after surgery at this writing.

## Discussion

Carcinoid tumors of the rectum are considered to be a frequent primary site [5]. One-half of all rectal carcinoids are discovered during anorectal examinations in asymptomatic patients. The remainders are found primarily by examinations of patients for symptoms (bleeding, constipation, rectal pain and so on). They are discovered most frequently in the 5th and 6th decades of life, with an equal gender distribution. Rectal carcinoid tumors usually occur singly, and the reported incidence of multiple carcinoid tumors is only 2% to 4.5% [6].

On the other hand, gastrointestinal ganglioneuromas may be classified into three major categories: diffuse ganglioneuromatosis, ganglioneuromatous polyposis, and polypoid ganglioneuromas [4]. Diffuse ganglioneuromatosis is a poorly demarcated nodular and diffuse intramural or transmural proliferation of ganglioneuromatous tissue elements involving the enteric plexuses. Transmural ganglioneuromatosis with the involvement of the myenteric plexus predominates in individuals with multiple endocrine neoplasia IIB (MEN IIB), whereas the involvement limited to the mucosa characterizes the disease in von Recklinghausen's disease [3]. Colonic adenocarcinoma has been described in association with diffuse ganglioneromatosis and ganglioneuromatous polyposis in a small number of cases [3]. However, no association with MEN IIB, von Recklinghausen's disease and adenocarcinoma has been observed in our patient. Localized gastrointestinal ganglioneuromatosis produces no characteristic symptoms and they are noted incidentally at endoscopy, surgery, or autopsy. Abdominal pain, obstruction, constipation, ileus, weight loss, and appendicitis are all considered to be related to the location and extent of the tumors. In our patient, gastrointestinal ganglioneuromatosis was not diagnosed before the operation.

Carcinoid tumors have been reported to be part of hereditary cancer syndrome [7]. The most common association is with the multiple endocrine neoplasia (MEN) syndrome. Although gastrointestinal ganglioneuromas have been especially reported to be associated with MEN IIB, carcinoid tumors are more frequently associated with MEN I [7]. Carcinoid and ganglioneuroma are both indicative of an overlapping syndrome of common neuroendocrine origin [8]. Patients with carcinoid tumors and ganglioneuromatosis should therefore be carefully observed for further manifestations of MEN syndrome.

The surgical treatment for carcinoid tumors is generally dictated to a degree, but not absolutely, by the size [1]. Tumors less than 1 cm in diameter are rarely associated with metastatic disease. Tumors measuring from 1.0 to 1.9 cm in diameter tend to show metastases in 10% of all cases. Lymph node or liver metastases are seen in from 80 to 100% of tumors measuring more than 2 cm in diameter. Rectal carcinoid tumors measuring less than 1 cm are usually treated by local excision including endoscopic techniques. In our case, the sizes of about 30 carcinod tumors were all less than 1 cm. The treatment of ganglioneuroma also consists of a local excision as these tumors usually exhibit a benign clinical behavior. However, several reports have shown that the incidence of lymph node metastasis is higher (10 to 22.7%) in multiple rectal carcinoid tumors measuring less than 1 cm than in isolated single tumors less than 1 cm [2]. Furthermore, we determined that a resection by an endoscopic operation was difficult, because preoperatively about 100 submucosal nodules were identified, including carcinoid tumors and ganglioneuromas, in the rectum. As a result, an abdominoperineal resection was performed.

## Conclusion

Although the optimal treatment for the multiple rectal carcinoids remains to be clearly established, it is believed that not all patients with multiple rectal carcinoids (measuring less than 1 cm in diameter) need to have a radical operation. However, the treatment plan for each case should be individualized and a careful follow-up is mandatory.

## Competing interests

The author(s) declare that they have no competing interests.

## Authors' contributions

MH, MK, NT, TK and JF carried out the surgical procedures; MH and JF contributed to the design of the study; HK gathered the data form the literature search; YI performed the histological analysis of all surgical specimens and provided histological sections as figures for the manuscript; TK revised and finally approved the manuscript for been published. All authors approved the final manuscript.
